# Correlations of *EZH2* and *SMYD3* gene polymorphisms with breast cancer susceptibility and prognosis

**DOI:** 10.1042/BSR20170656

**Published:** 2018-02-02

**Authors:** Shao-Jun Ma, Yan-Mei Liu, Yue-Lang Zhang, Ming-Wei Chen, Wei Cao

**Affiliations:** 1 Department of Radiology, Shaanxi Provincial People’s Hospital, Xi’an 710068, P.R. China; 2 The Department of West Yard Ward 2, Shaanxi Provincial People’s Hospital, Xi’an 710068, P.R. China; 3 Department of Radiology, The First Affiliated Hospital of Xi’an JiaoTong University, Xi’an 710061, P.R. China; 4 Department of Respiration, The First Affiliated Hospital of Xi’an JiaoTong University, Xi’an 710061, P.R. China; 5 Department of Surgical Oncology, Shaanxi Provincial People’s Hospital, Xi’an 710068, P.R. China

**Keywords:** Breast cancer, EZH2, Polymorphism, Prognosis, SMYD3, Susceptibility

## Abstract

The aim of the present study was to investigate the correlation of enhancer of Zeste homolog 2 (*EZH2*) and SET and MYND domain containing 3 (*SMYD3*) gene polymorphisms with breast cancer susceptibility and prognosis. A total of 712 patients with breast cancer and 783 healthy individuals were selected. Normal breast epithelial cells MCF-10A and breast cancer cells MCF-7, MDA-MB-231, T47D, and Bcap-37 were cultured. Polymerase chain reaction (PCR)-restriction fragment length polymorphism method was applied for genotyping. Reverse-transcription quantitative PCR (RT-qPCR) and Western blotting were used to examine *EZH2* and *SMYD3* expression in breast cancer tissues and cells. The risk factors and prognostic factors for breast cancer were estimated. The C allele of *EZH2* rs12670401 (odds ratio (OR) =1.255, 95% confidence interval (95% CI): 1.085–1.452), T allele of *EZH2* rs6464926 (OR =1.240, 95% CI: 1.071–1.435), and three alleles of *SMYD3* variable number of tandem repeats (VNTRs) (OR =1.305, 95% CI: 1.097–1.552) could increase susceptibility to breast cancer. Combined genotypes of *EZH2* rs12670401 (TC + CC) and *EZH2* rs6464926 (CT + TT) were associated with breast cancer susceptibility. Breast cancer tissues had higher *EZH2* and *SMYD3* expression. *EZH2* rs12670401, *EZH2* rs6464926, age of menarche, and menopausal status were associated with breast cancer susceptibility. Patients with TT genotype of *EZH2* rs12670401 or with CC genotype of *EZH2* rs6464926 had higher overall survival (OS). *EZH2* rs12670401, *EZH2* rs6464926, and clinical staging were independent prognostic factors for breast cancer. *SMYD3* VNTR polymorphism exhibited no association with susceptibility and prognosis. *EZH2* rs12670401 and rs6464926 polymorphisms, *EZH2* and *SMYD3* expression, clinical staging, lymph node metastasis, human epidermal growth factor receptor-2 (HER2) status, and metastasis may be correlated with breast cancer susceptibility and prognosis.

## Introduction

Breast cancer, the most common malignant tumor in the world and second common malignant tumor in China, ranks sixth as the cause of female tumor deaths [1]. The hazard and developing trend of breast cancer is closely related to age, lifestyle, economic development, and environmental change, and the mortality of breast cancer in Chinese females shows a gradual upward trend in recent years [2]. Surgical therapy is currently the main treatment for breast cancer, including hormonal therapy, chemotherapy, radiotherapy, and molecular targetted therapy [3]. Many molecular targets have been identified in breast cancer: trastuzumab and lapatinib target the human epidermal growth factor receptor-2 (HER2) and are approved drugs for the treatment of metastatic breast cancer [4]. In addition, the expression abnormality of relative genes has been proved to be involved in the incidence of breast cancer and may cause the proliferation, invasion, recurrence, and metastasis of tumors [5]. Researches in genetic polymorphisms of breast cancer can provide a theoretical basis for breast cancer prevention, diagnosis, therapy, and prognosis [6–8].

Epigenetic histone modification, a kind of genetic modification, plays a role in the regulation of gene expression by affecting the affinity of histone, DNA duplexes, binding of transcription factors, and DNA structural gene promoters [9]. Histone methylation is a member of the histone modification with histone methyltransferase activity, wherein SET and MYND domain containing 3 (*SMYD3*) and enhancer of Zeste homolog 2 (*EZH2*) are reported to be involved in the development of multiple cancers and play important roles in transcriptional regulation [10]. It was found that *EZH2* and *SMYD3* could stimulate growth and increase invasion of multiple tumors [11,12]. In recent years, gene polymorphisms have been reported to play important roles in tumor development and progression [13]. *EZH2* polymorphism has a significant influence on colorectal cancer (CRC) susceptibility in the Han Chinese population and plays an important role in the pathogenesis and prediction of CRC [14]. Polymorphisms (rs12670401 and rs6464926) of *EZH2* were identified to be significantly associated with the risk of gastric cancer and C allele of *EZH2* rs12670401 and T allele of EZH2 rs6464926 showed strong associations with increased gastric cancer susceptibility [15]. A variable number of tandem repeats (VNTRs) polymorphism in the *SMYD3* promoter region is a risk factor for familial breast cancer and esophageal squamous cell carcinoma [16,17]. However, the correlation of *EZH2* and *SMYD3* polymorphisms with breast cancer susceptibility and prognosis has not yet been reported. Therefore, the present study aims to investigate the correlation of *EZH2* and *SMYD3* gene polymorphisms with breast cancer susceptibility and prognosis, in order to provide a certain theoretical basis for clinical application in the diagnosis and prognosis of breast cancer, as well as a reference for individualized therapy of breast cancer.

## Materials and methods

### Ethics statement

The experimental procedures were approved by the Human Ethics Committee of Shaanxi Provincial People’s Hospital and were performed in accordance with the ethical standards laid down in the 1964 Declaration of Helsinki.

### Study subjects

From August 2010 to December 2012, 712 patients with breast cancer (all females, mean age: 49.88 ± 13.14 years) who were admitted in Shaanxi Provincial People’s Hospital and the co-operative hospital (First Hospital of Xi’an Jiao Tong University) were randomly selected as a case group. The inclusion criteria were as follows: patients received X-ray mammography and were confirmed with pathological examination as having breast cancer. A total of 783 cancer-free healthy people with no sibship with involved patients (mean age: 45.51 ± 11.21 years) who took physical examination in Shaanxi Provincial People’s Hospital in the same period were classified as the control group. The exclusion criteria were as follows: (i) patients with extensive metastasis; (ii) patients with tumor history in other organs; (iii) patients with systemic failure, systemic lupus erythematosus, or other autoimmune diseases; (iv) patients with recent trauma, surgeries, lymph nodes, or other malignant cancer; (v) patients who did not co-operate with surgery and investigation; (vi) patients without detailed clinical data or follow-up data. All 712 samples of breast cancer tissues not treated with radiotherapy or chemotherapy before operation were collected. All 783 normal adjacent tissues were extracted at least 4 cm from the cancer tissues. All tissues were stored at −80°C after cryopreservation.

### Cell culture

Normal breast epithelial cells MCF-10A and breast cancer cells MCF-7, MDA-MB-231, T47D, and Bcap-37 were purchased from American Type Culture Collection (ATCC, Manassas, VA, U.S.A.). After resuscitation, MCF-10A was cultured in Dulbecco’s modified Eagle’s medium (DMEM)/F12 medium containing 5% horse serum, 10 g/ml insulin, 20 ng/ml epidermal growth factor (EGF), 100 ng/ml cholera toxin, and 0.5 g/ml hydrocortisone; MDA-MB-231 was cultured in RPMI1640 medium containing 10% FBS, 100 U/ml penicillin, 100 Ug/ml streptomycin, and 2 mol/l glutamine; MCF-7 was cultured in DMEM containing 10% FBS, 100 U/ml penicillin, 100 Ug/ml streptomycin, and 2 mol/l glutamine; T47D was cultured in DMEM containing 20% FBS, 100 U/ml penicillin, 100 Ug/ml streptomycin, and 2 mol/l glutamine; Bcap-37 was cultured in RPMI1640 medium containing 20% FBS, 100 U/ml penicillin, 100 Ug/ml streptomycin, and 2 mol/l glutamine. All five kinds of cells were cultured in 5% CO_2_ incubation at 37°C with saturated humidity. The culture medium was changed every 2–3 days. When the cells grew well and reached 80–90% confluence, they were collected.

### Peripheral venous blood collection and genomic DNA extraction

Fasting peripheral blood was collected in the case and control groups for EDTA anticoagulant and frozen preservation. Genomic DNA was extracted with Blood Genome DNA Extraction Kit (Takara Biotechnology Ltd., Dalian, China) after blood sampling. Under high-salt state, the DNA was specifically adsorbed by a silicone membrane; under the condition of low salt or aqueous solution, the DNA was eluted. The DNA concentration was adjusted to 100 ng/μl and preserved in a refrigerator at −20°C.

### Polymerase chain reaction-restriction fragment length polymorphism (PCR-RFLP)

PCR-RFLP was used to detect and analyze polymorphic sites of genotypes and the prime 5.0 software was applied for primer design. All the primers were synthesized by Shanghai Sangon Biological Engineering Technology & Services Co., Ltd. (Shanghai, China) and the primer sequences are shown in Table 1 and Table 2.

**Table 1:** Primer sequences of SNP for *EZH2* and *SMYD3*

SNP	Primer sequence	Temperature	Time	Circle
*EZH2rs12670401*	F: 5′-ACGTTGGATGTACTTGGTTTAGATTGCCTG-3′	66°C	45 s	30
	R: 5′-ACGTTGGATGATGGACTAGGTGGTTCAAAC-3′			
*EZHrs6464926*	F: 5′-ACGTTGGATGAGAATTGCCAGGAGATCTCT-3′	58°C	45 s	40
	R: 5′-ACGTTGGATGTTCCTTCATTGCCTGTTGCC-3′			
*SMYD3VNTR*	F: 5′-CGCCTGTCTTCTGCGCAGTCG-3′	56.5°C	45 s	40
	R: 5′-CACCTTCAGCGGCTCCATCCTC-3			

Abbreviations: F, forward; R, reverse; SNP, single nucleotide polymerase.

**Table 2:** Primer sequences of *EZH2* and *SMYD3*

Genes	Sequences (5′–3′)
*EZH2*	F: 5′-AATCAGAGTACATGCGACTGAGA-3′
	R: 5′-GCTGTATCCTTCGCTGTTTCC-3′
*SMYD3*	F: 5′-CGCGTCGCCAAATACTGTAGT-3′
	R: 5′-CAAGAAGTCGAACGGAGTCTG-3′
*GAPDH*	F: 5′-GGAGCGAGATCCCTCCAAAAT-3′
	R: 5′-GGCTGTTGTCATACTTCTCATGG-3′

Abbreviations: F, forward; GAPDH, glyceraldehyde phosphate dehydrogenase; R, reverse.

The PCR reaction system of *EZH2* rs12670401: the total reaction system was 25 μl, including 12.5 μl of 2× Master Mix, 1 μl (10 mM) of upstream and downstream primers, respectively, 1 μg of DNA template, and 25 μl of ddH_2_O. PCR conditions: 2 min of predenaturation at 66°C, 30 cycles of 30 s of denaturation at 94°C, 45 s of annealing at 66°Cand 30 s of extension at 72°C, and finally 7 min of extension at 72°C.

The PCR reaction system of *EZH2* rs6464926: the total reaction system was 50 μl, including 5 μl of 10× buffer, 4 μl (25 mM) of MgCl_2_, 1 μl (10 mM) of dNTPs, 1 μl (25 pmol/l) of upstream and downstream primers, respectively, 10 μl of DNA template, and 27 μl of ddH_2_O. PCR conditions: 2 min of predenaturation at 95°C, 40 cycles of 1 min of denaturation at 95°C, 45 s of annealing at 58°Cand 1 min of extension at 72°C, and finally 2 min of extension at 72°C.

The PCR reaction system of *SMYD3* VNTR: the total reaction system was 50 μl, including 5 μl of 10× buffer, 4 μl (25 mM) of MgCl_2_, 1 μl (10 mM) of dNTPs, 1 μl (25 pmol/l) of upstream and downstream primers, respectively, 10 μl of DNA template, and 27 μl of ddH_2_O. PCR conditions: 2 min of predenaturation at 94°C, 40 cycles of 20 s of denaturation at 94°C, 45 s of annealing at 56.5°C, and 30 s of extension at 72°C, and finally 3 min of extension at 72°C.

**Figure 1:**
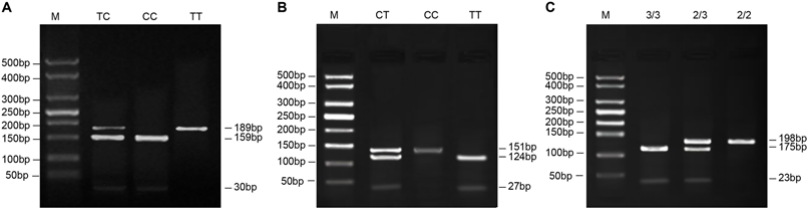
Electrophoresis maps of *EZH2* rs12670401, *EZH2* rs6464926, and *SMYD3*VNTR gene polymorphisms (**A**) Electrophoresis maps of *EZH2* rs12670401 gene polymorphisms. M, DNA marker; TC, TC heterozygotes digestion products, 189, 159, 30 bp; CC, CC homozygotes digestion products, 159 and 30 bp; TT, TT homozygotes digestion product, 189 bp. (**B**) Electrophoresis map of *EZH2* rs6464926 gene polymorphisms. M, DNA marker; CC, CC homozygotes digestion products, 151 bp; TT, TT homozygotes digestion products, 124 and 27 bp; CT, CT heterozygotes digestion products, 124, 151, and 27 bp. (**C**) Electrophoresis map of *SMYD3* VNTR gene polymorphisms. M, DNA marker; 3/3, 3/3 homozygote digestion products, 175 and 23 bp; 2/3, 2/3 heterozygotes digestion products, 198, 175, and 23 bp; 2/2, 2/2 homozygotes digestion products, 198 bp.

The PCR amplification product was digested with restricted enzyme. The total reaction system was 20 μl, including 17 μl of PCR amplification product, 2 μl of 10× reaction buffer, and 1 μl (10 U/μl) of matching buffer, followed by addition of incision enzyme (1 μl) for overnight digestion at 37°C. The enzyme digestion products were evaluated by 3.5% agarose gel electrophoresis (120 V, 40 min) and Ethidium Bromide staining. Moreover, the DNA bends (Figure 1) were observed and detected under a UV lamp. Ten percent samples randomly collected were sequenced from both directions by Shanghai Sangon Biological Engineering Technology & Services Co., Ltd. (Shanghai, China) to verify the results of PCR detection. Genotypes were determined according to the enzyme map.

### Reverse-transcription quantitative PCR

MiRNeasy Mini Kit (Qiagen Company, Hilden, Germany) was used to extract total RNA from tissues or cells. The absorbance and purity of RNA were determined by UV spectrophotometer at 260 and 280 nm. If the ratio of optical density (OD)260/OD280 was between 1.7 and 2.1, the purity was higher and could be used in subsequent experiments. The cDNA template was synthesized by reverse transcription in PCR amplification apparatus (ABI). ABI7500 quantitative PCR (ABI Company, Oyster Bay, NY, U.S.A.) was used to perform the reverse-transcription quantitative PCR (RT-qPCR) experiment, and the reaction conditions were as follows: 10 min of predenaturation at 95°C, 10 s of denaturation at 95°C, 20 s of annealing at 60°C, 34 s of extension at 72°C, totalling 40 cycles. The reaction system included SYBR Premix Ex Taq™ II 10 µl, PCR forward primer (10 µM) 0.8 µl, PCR reverse primer (10 µM) 0.8 µl, ROX Reference Dye 0.4 µl, cDNA template 2.0 µl, and sterilized distilled water 6.0 µl. The glyceraldehyde phosphate dehydrogenase (GAPDH) was used as a reference, and 2^−ΔΔ*C*^_t_ indicated the ratio of target gene expression between the experiment group and the control group. The formula was as follows: ΔΔ*C*_T_ = Δ*C*_t experiment group_ – Δ*C*_t control group_, and Δ*C*_t_ = *C*_t target gene_ – *C*_t__GAPDH._ *C*_t_ was the cycle numbers in which the fluorescence intensity reached the set threshold when the amplification was at logarithmic growth phase [18].

### Western blotting

The cancer tissues and adjacent tissues were added with liquid nitrogen and ground until the tissues were homogeneous. Then, the tissues were added with protein lysate (Beijing Solarbio Science & Technology Co., Ltd., Beijing, China), centrifuged at 12000 rev/min at 4°C for 20 min, and the supernatant was obtained and stored for further use. The cells were collected, lysed, and centrifuged, and the total protein was collected. The total protein concentration was measured by BCA Kit (Thermo Fisher Scientific, Carlsbad, California, U.S.A.). After taking the total protein of the cell and separating by SDS/PAGE (12% gel), the protein was transferred on to PVDF membrane and sealed by skimmed milk at room temperature for 1 h. The following primary antibodies were added – EZH2 (1:1000, ab186006), SMYD3 (1:2000, ab187149), and GAPDH (1:1000, ab8245) – and cultured at 4°C overnight. All antibodies were purchased from Abcam Inc. (Cambridge, MA, U.S.A.). The membrane was washed three times to remove the primary antibodies, and added with second antibody and cultured at room temperature for 1 h. The membrane was washed three times again and ECL reagent was applied to reveal Western blotting bands. Using GAPDH as a reference, the relative expression of protein was analyzed by Western blotting image (ImageJ2x software).

### Follow-up

With approval of the patients and their families, follow-up was carried out on a regular basis by telephone, outpatient, letters, or clinical data consulting for data statistics. The follow-up duration was 60 months and the follow-up rate was 100%. The patients’ condition changes were informed regularly through follow-up feedback and the clinical pathological data and survival rate were collected for statistical analysis.

### Statistical analysis

SPSS 19.0 statistical software (IBM Corp. Armonk, NY, U.S.A.) was applied for data processing. Hardy–Weinberg equilibrium was used to test the population representativeness. A *P*-value ≥0.05 indicated that the sample reached genetic equilibrium with good population representativeness. Univariate and multivariate logistic regression analyses were applied to calculate odds ratio (OR) and 95% confidence interval (95% CI), to estimate the strength of association between the mutation of each polymorphic locus and breast cancer. The enumeration data were represented as ratio or rate, and compared by *χ*^2^ test. The measurement data were represented as mean ± S.D., and compared by *t* test, with *P*<0.05 as statistically significant. *P* was a two-sided test, and *P*<0.05 was considered to be statistically significant.

## Results

### Baseline characteristics of study subjects

No significant difference in age (*P*>0.05), but obvious differences in age of menarche and menopausal status were found between the case and control groups (both *P*<0.05). In the case group, patients with age over 40 years accounted for 83.01%; 75.28% patients were positive in estrogen receptor (ER^+^) and 76.41% were positive in progesterone receptor (PR^+^); 91.85% patients were in clinical stage I or II, and 8.15% (58 cases) were in stage III or IV; 377 cases (52.94%) had lymph node metastasis (Table 3).

**Table 3:** Baseline characteristics of the study subjects between the case and control groups

Groups	Case group, *n* (%)	Control group, *n* (%)	*P*
Total	712	783	
Age (years)			
≥40	591 (83.01 )	673 (85.95)	0.132
<40	121 (16.99)	110 (14.05)	
Age of menarche (years)			
≥13	495 (69.52)	477 (60.92)	<0.001
<13	217 (30.48)	306 (39.08)	
Menopausal status			
Premenopause	81 (11.38)	292 (37.29)	<0.001
Postmenopause	631 (88.62)	491 (62.71)	
Tumor size (cm)			
≤2.0	323 ( 45.37)		
2.1–4	329 (46.21)		
>4	60 (8.43)		
Pathological type			
IDC	618 (86.80)		
ILC	31 (4.35)		
Other	63 (8.85)		
Lymph node metastasis			
Negative	334 (46.91)		
Positive	378 (53.09)		
Immunohistochemical indexes			
ER (−)	176 (24.72)		
ER (+)	536 (75.28)		
PR (−)	168 (23.60)		
PR (+)	544 (76.41)		
HER2 status			
Negative	361 (50.70)		
Positive	351 (49.30)		
FDRs with breast cancer			
Yes	251 (35.25)		
No	461 (64.75)		
Clinical staging	654 (91.85)		
I/II			
III/IV	58 (8.15)		
Subtypes of breast cancer			
Triple negative	133 (18.68)		
Non-triple negative	579 (81.32)		
Metastasis situation			
Distant metastasis	154 (21.63)		
Primary lesion	558 (78.37)		

Abbreviations: FDR, first-degree relative; IDC, invasive ductal carcinoma; ILC, invasive lobular carcinoma.

### Electrophoresis map of *EZH2* and *SMYD3* gene polymorphisms

The amplified fragment length of *EZH2* rs12670401 was 189 bp, wherein there were no fragments in wild-type (TT-type), two fragments (159 and 30 bp) in homozygous mutant (CC type), and three fragments (189, 159, and 30 bp) in heterozygous mutant (TC type) after HaeIII digestion (Figure 1A). The amplified fragment length of *EZH2* rs6464926 was 151 bp, wherein there were two fragments (124 and 27 bp) in homozygous mutant (TT type), three fragments (124, 151, and 27 bp) in heterozygous mutant (CT type), and a fragment (151 bp) in wild-type (CC type) after NdeI digestion (Figure 1B). The amplified fragment length of *SMYD3* VNTR was 198 bp, wherein there were two fragments (175 and 23 bp) in homozygous mutant (3/3 type), three fragments (198, 175, and 23 bp) in heterozygous mutation (2/3 type), and a fragment (198 bp) in wild genotype (2/2 type) after HinfI digestion (Figure 1C).

### Frequency distribution of EZH2 and SMYD3 genotypes and alleles

The genotypes and alleles frequency distribution of *EZH2* rs12670401, *EZH2* rs6464926, and *SMYD3* VNTR polymorphisms in the case and control groups conformed to Hardy–Weinberg equilibrium with population representativeness (*P*>0.05).

In the case and control groups, there were significant differences in the allele frequency (*χ*^2^ =9.356, *P*=0.002) and genotype frequency (*χ*^2^ =12.22, *P*<0.001) of *EZH2* rs12670401, and C allele could increase the susceptibility to breast cancer (OR =1.255, 95% CI: 1.085–1.452, *P*=0.002). There was an obvious difference in allele frequency (*χ*^2^ =8.286, *P*=0.004) and genotype frequency (*χ*^2^ =8.972, *P*=0.003) of *EZH2* rs6464926, and T allele could increase the susceptibility to breast cancer (OR =1.240, 95% CI: 1.071–1.435, *P*=0.004). There was a distinctive difference in allele frequency (*χ*^2^ =9.1, *P*=0.034) and genotype frequency (*χ*^2^ =39.971, *P*<0.001) of *SMYD3* VNTR, and 3 allele could increase the susceptibility to breast cancer (OR =1.305, 95% CI: 1.097–1.552, *P*=0.003) (Table 4).

**Table 4:** Genotype and allele frequency of *EZH2* rs12670401, *EZH2* rs6464926, and *SMYD3* VNTR between the case and control groups

Genotype/allele	Case group, *n* (%)	Control group, *n* (%)	*χ*^2^	*P*	OR (95% CI)
*EZH2* rs12670401					
TT	232 (32.58)	290 (37.04)			1
TC	323 (45.37)	372 (47.51)	0.495	0.482	1.085 (0.864–1.363)
CC	157 (22.05)	121 (15.45)	12.22	<0.001	1.687 (1.257–2.265)
TC + CC	480 (67.42)	493 (62.96)	3.254	0.071	1.217 (0.983–1.507)
T	787 (55.27)	952 (60.79)			1
C	637 (44.73)	614 (39.21)	9.356	0.002	1.255 (1.085–1.452)
*EZH2* rs6464926					
CC	243 (34.13)	305 (38.95)			1
CT	327 (45.93)	365 (46.62)	1.044	0.307	1.124 (0.898–1.408)
TT	142 (19.94)	113(14.43)	8.972	0.003	1.577 (1.169–2.127)
CT + TT	469 (65.87)	478 (61.05)	3.737	0.053	1.232 (0.997–1.521)
C	813 (57.09)	975 (62.26)			1
T	611 (42.91)	591 (37.74)	8.286	0.004	1.240 (1.071–1.435)
*SMYD3*VNTR					
2/2	427 (59.97)	483 (61.69)			1
2/3	222 (31.18)	289 (36.91)	1.596	0.207	0.869 (0.699–1.081)
3/3	63 (8.85)	11 (1.40)	39.971	<0.001	6.478 (3.369–12.456)
2/3 + 3/3	285 (40.03)	300 (38.31)	0.459	0.498	1.075 (0.873–1.323)
2	1076 (75.56)	1255 (80.14)			1
3	348 (24.44)	311 (19.86)	9.1	0.003	1.305 (1.097–1.552)

### Interaction of EZH2 and SMYD3 polymorphic loci with the susceptibility to breast cancer

As shown in Table 5, the combined genotype of *EZH2* rs12670401 (TC + CC) and *EZH2* rs6464926 (CT + TT) was significantly associated with the susceptibility to breast cancer (OR =1.465, 95% CI: 1.055–2.036, *P*=0.022). There was no statistical significance in the interaction of other genotypes with the susceptibility to breast cancer (all *P*>0.05).

**Table 5:** Correlation of interaction of *EZH2* rs12670401, *EZH2* rs6464926, and *SMYD3* VNTR gene polymorphisms with breast cancer susceptibility in the case and control groups

Combined genotype	Case group, *n* (%)	Control group, *n* (%)	OR	95% CI	*P*
*EZH2* rs12670401 (TC + CC)/*EZH2* rs6464926 (CT + TT)	316 (44.41)	303 (38.76)	1.465	1.055–2.036	0.022
*EZH2* rs12670401 (TC + CC)/*EZH2* rs6464926 (CC)	164 (23.01)	190 (24.20)	1.213	0.849–1.732	0.288
*EZH2* rs12670401 (TT)/*EZH2* rs6464926 (CT + TT)	153 (21.46)	179 (22.80)	1.201	0.838–1.722	0.319
*EZH2* rs12670401 (TT)/*EZH2* rs6464926 (CC)	79 (11.12)	111 (14.24)		Reference	
*EZH2* rs12670401 (TC + CC)/ *SMYD3*VNTR(2/3 + 3/3)	192 (26.99)	99 (12.69)	1.312	0.918–1.874	0.136
*EZH2* rs12670401 (TC + CC)/ *SMYD3*VNTR (2/2)	288 (40.43)	160 (20.44)	1.217	0.879–1.685	0.236
*EZH2* rs12670401 (TT)/ *SMYD3*VNTR(2/3 + 3/3)	93 (13.04)	58 (7.47)	1.084	0.713–1.650	0.705
*EZH2* rs12670401 (TT)/ *SMYD3*VNTR (2/2)	139 (19.54)	94 (12.02)		Reference	
*SMYD3*VNTR(2/3 + 3/3)/*EZH2* rs6464926 (CT + TT)	188 (26.37)	185 (44.82)	1.288	0.957–1.733	0.095
*SMYD3*VNTR (2/3 + 3/3)/*EZH2* rs6464926 (CC)	97 (13.66)	115 (27.99)	1.069	0.756–1.512	0.707
*SMYD3*VNTR (2/2)/*EZH2* rs6464926 (CT + TT)	281 (39.50)	297 (72.17)	1.195	0.911–1.567	0.198
*SMYD3*VNTR (2/2)/*EZH2* rs6464926 (CC)	146 (20.47)	186 (45.07)		Reference	

Reference, 95% CI is 1.

### Expression of EZH2 and SMYD3 is higher in cancer tissues than in adjacent tissues

**Figure 2:**
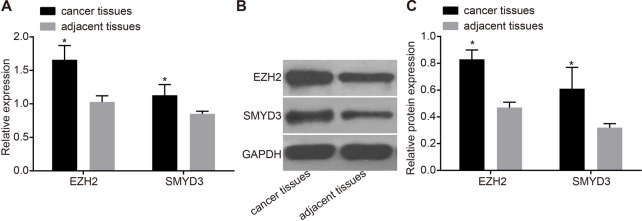
Expression of EZH2 and SMYD3 in cancer tissues and adjacent tissues (**A**) mRNA expression of EZH2 and SMYD3 in cancer tissues and adjacent tissues. (**B**) Western blotting. (**C**) Protein expression of EZH2 and SMYD3 in cancer tissues and adjacent tissues.

The mRNA and protein expression of EZH2 and SMYD3 in cancer tissues and adjacent tissues were detected by RT-qPCR and Western blotting. The results showed that the mRNA and protein expression of EZH2 and SMYD3 in cancer tissues were higher than those in adjacent tissues (*P*<0.05) (Figure 2). The results suggested that EZH2 and SMYD3 were in high expression in breast cancer tissues.

### Expression of EZH2 and SMYD3 is higher in breast cancer cells

**Figure 3:**
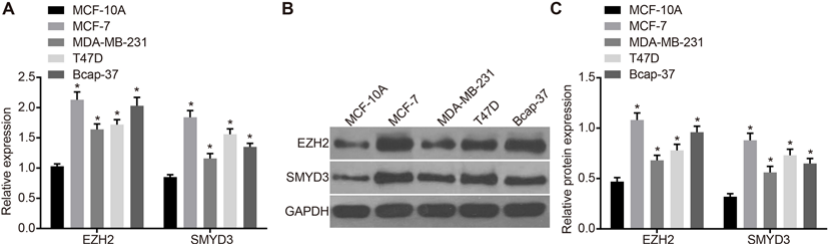
mRNA and protein expression of EZH2 and SMYD3 in breast cancer cells (**A**) mRNA expression of EZH2 and SMYD3 in breast cancer cells. (**B**) Immunoblotting map. (**C**) Protein expression of EZH2 and SMYD3 in breast cancer cells; *P<0.05, compared with MCF-10A cells.

The mRNA and protein expression of EZH2 and SMYD3 in normal breast epithelial cells MCF-10A and breast cancer cells MCF-7, MDA-MB-231, T47D, and Bcap-37 were detected by RT-qPCR and Western blotting. The results showed that the mRNA and protein expression of EZH2 and SMYD3 in breast cancer cells MCF-7, MDA-MB-231, T47D, and Bcap-37 were higher than those in normal breast epithelial cells MCF-10A (*P*<0.05) (Figure 3). The results suggested that EZH2 and SMYD3 were in high expression in breast cancer cells.

### Relationship between mRNA expression of EZH2 and SMYD3 and clinicopathological features

The results showed that mRNA expression of EZH2 had no statistical significance with patients’ age, age of menarche, menopausal status, tumor size, pathological type, immunohistochemical indexes, and first-degree relatives (FDRs) with breast cancer (*P*>0.05), but had statistical significance with lymph node metastasis, HER2 status, clinical staging, subtypes of breast cancer, and metastasis situation. mRNA expression of SMYD3 had no statistical significance with patients’ age, age of menarche, menopausal status, tumor size, pathological type, immunohistochemical indexes, FDRs with breast cancer, and clinical staging (*P>*0.05), but had statistical significance with lymph node metastasis, HER2 status, subtypes of breast cancer, and metastasis situation (Table 6).

**Table 6:** The relationship between mRNA expression of EZH2 and SMYD3 and clinicopathological features

Pathological characteristics	Case	*EZH2* mRNA expression	*P*	*SMYD3* mRNA expression	*P*
Age (years)			0.623		0.206
≥40	591	1.66 ± 0.20		1.13 ± 0.16	
<40	121	1.67 ± 0.22		1.15 ± 0.15	
Age of menarche (years)			0.553		0.999
≥13	495	1.66 ± 0.21		1.13 ± 0.15	
<13	217	1.67 ± 0.20		1.13 ± 0.17	
Menopausal status			0.677		0.113
Premenopause	81	1.67 ± 0.23		1.16 ± 0.16	
Postmenopause	631	1.66 ± 0.20		1.13 ± 0.16	
Tumor size (cm)			0.372		0.197
≤2.0	323	1.67 ± 0.19		1.14 ± 0.16	
2.1–4	329	1.66 ± 0.22		1.13 ± 0.16	
>4	60	1.63 ± 0.20		1.10 ± 0.16	
Pathological type			0.368		0.779
IDC	618	1.66 ± 0.20		1.13 ± 0.15	
ILC	31	1.66 ± 0.20		1.15 ± 0.15	
Other	63	1.66 ± 0.23		1.13 ± 0.19	
Lymph node metastasis			<0.001		<0.001
Negative	335	1.61 ± 0.19		1.09 ± 0.14	
Positive	377	1.71 ± 0.21		1.17 ± 0.17	
Immunohistochemical indexes			0.392		0.433
ER (−)	176	1.66 ± 0.21		1.15 ± 0.15	
ER (+)	536	1.66 ± 0.21		1.13 ± 0.16	
PR (−)	168	1.66 ± 0.21		1.14 ± 0.15	
PR (+)	544	1.66 ± 0.20		1.13 ± 0.16	
HER2 status			<0.001		<0.001
Negative	361	1.62 ± 0.18		1.10 ± 0.14	
Positive	351	1.71 ± 0.22		1.16 ± 0.17	
FDRs with breast cancer			0.531		0.426
Yes	251	1.67 ± 0.21		1.14 ± 0.16	
No	461	1.66 ± 0.20		1.13 ± 0.16	
Clinical staging			0.004		0.154
I/II	654	1.66 ± 0.20		1.13 ± 0.15	
III/IV	58	1.74 ± 0.25		1.16 ± 0.19	
Subtypes of breast cancer			<0.001		<0.001
Triple negative	133	1.74 ± 0.25		1.18 ± 0.17	
Non-triple negative	579	1.65 ± 0.19		1.12 ± 0.15	
Metastasis situation			<0.001		<0.001
Distant metastasis	154	1.74 ± 0.21		1.19 ± 0.19	
Primary lesion	558	1.64 ± 0.20		1.12 ± 0.14	

Abbreviations: IDC, invasive ductal carcinoma; ILC, invasive lobular carcinoma.

### mRNA expression of EZH2 and SMYD3 in patients with different genotypes

The results showed that mRNA expression of EZH2 in CC genotype carriers of *EZH2* rs12670401 was higher than that in TT and TC genotype carriers (*P*<0.05); mRNA expression of EZH2 in C allele carriers of *EZH2* rs12670401 was higher than that in T allele carriers (*P*<0.05); mRNA expression of EZH2 in TT genotype carriers of *EZH2* rs6464926 was higher than that in CC and CT genotype carriers (*P*<0.05); mRNA expression of EZH2 in T allele carriers of *EZH2* rs6464926 was higher than that in C allele carriers (*P*<0.05). mRNA expression of SMYD3 in 3/3 genotype carriers of *SMYD3*VNTR was higher than that in 2/3 and 2/2 genotype carriers, but the difference was not statistically significant (*P*>0.05). The difference in the mRNA expression of SMYD3 between the different allele carriers in *SMYD3*VNTR was not statistically significant (*P*>0.05) (Table 7).

**Table 7:** mRNA expression of EZH2 and SMYD3 in patients with different genotypes

Gene loci	Case	mRNA expression	*P*
*EZH2* rs12670401			
TT	232	1.63 ± 0.18	0.01
TC	323	1.67 ± 0.21	
CC	157	1.69 ± 0.22	
T	787	1.65 ± 0.20	0.007
C	637	1.68 ± 0.22	
*EZH2* rs6464926			
CC	243	1.64 ± 0.18	0.02
CT	327	1.66 ± 0.22	
TT	142	1.70 ± 0.20	
C	813	1.65 ± 0.21	0.009
T	611	1.68 ± 0.22	
*SMYD3*VNTR			
2/2	427	1.13 ± 0.16	0.631
2/3	222	1.13 ± 0.15	
3/3	63	1.15 ± 0.17	
2	1076	1.13 ± 0.16	0.304
3	348	1.14 ± 0.15	

### Univariate and multivariate logistic regression analyses of risk factors for breast cancer

Multivariate logistic regression analysis was carried out with breast cancer as a dependent variable, and different genotypes (*EZH2* rs12670401, *EZH2* rs6464926, and *SMYD3* VNTR) in the case and control groups and baseline characteristics (age, age of menarche, and menopausal status) as independent variables. As shown in Table 8, *EZH2* rs12670401 and *EZH2* rs6464926 polymorphisms, age of menarche, and menopausal status were risk factors for breast cancer (all *P*<0.05), but age was not the risk factor of breast cancer (*P*>0.05).

**Table 8:** Multivariate logistic regression analysis of risk factors for breast cancer patients

	B	S.E.M.	OR	95% CI	*P*
*EZH2* rs12670401	0.697	0.095	2.007	1.666–2.418	<0.001
*EZH2* rs6464926	0.236	0.116	1.266	1.008–1.590	0.042
*SMYD3*VNTR	−0.173	0.118	1.223	0.667–1.060	0.143
Age	−0.186	0.151	0.830	0.618–1.115	0.216
Age of menarche	0.444	0.117	1.558	1.238–1.962	<0.001
Menopausal status	1.590	0.148	4.901	3.664–6.557	<0.001

Abbreviation: B, regression coefficient.

### Association of gene polymorphisms and mRNA expression of EZH2 and SMYD3 with breast cancer prognosis

**Figure 4:**
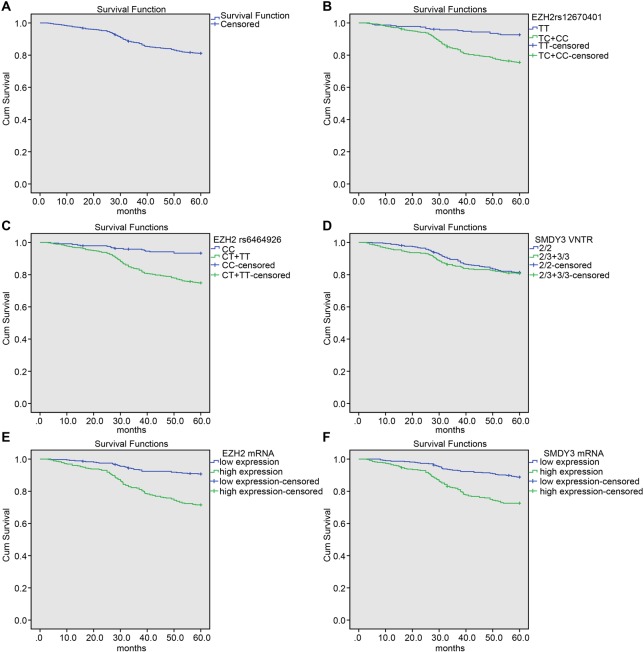
Correlation of *EZH2* rs12670401, *EZH2* rs6464926, and *SMYD3*VNTR gene polymorphisms with the OS of patients with breast cancer (**A**) OS curve for patients with breast cancer. (**B**) Survival curve for patients with *EZH2* rs12670401. (**C**) Survival curve for patients with *EZH2* rs6464926. (**D**) Survival curve for patients with *SMYD3* VNTR genotypes. (**E**) Survival curve for mRNA expression of EZH2. (**F**) Survival curve for mRNA expression of SMYD3.

The overall survival (OS) after 24, 36, 48, and 60 months of the patients were 95.23, 87.76, 84.13, and 81.18%, respectively (Figure 4). The association between polymorphisms and breast cancer prognosis was analyzed by Kaplan–Meier survival, and the result showed that the OS of patients with TT genotype was higher than those with TC + CC genotype in *EZH2* rs12670401 locus, and the OS of the patients with CC genotype was higher than those with CT + TT genotype in *EZH2* rs6464926 locus (both *P*<0.05); the OS of the patients with 2/2 genotype was higher than those with 2/3 + 3/3 genotype in *SMYD3* VNTR locus, but the difference was not statistically significant (*P*>0.05) (Figure 4). Patients were allocated into EZH2 high expression and EZH2 low expression, SMYD3 high expression, and SMYD3 low expression with the median of *EZH2* and *SMYD3*mRNA expression as boundary. The Kaplan–Meier survival analysis showed that the OS of patients with EZH2 high expression was lower than those with EZH2 low expression (*P*<0.05), and the OS of patients with SMYD3 high expression was lower than those with SMYD3 low expression (*P*<0.05).

Result of Cox multivariate analysis declared that *EZH2* rs12670401 and *EZH2* rs6464926 genotypes, mRNA and protein expression of EZH2 and SMYD3, clinical staging, lymph node metastasis, HER2 status, and metastasis situation were independent prognostic factors for the survival rate of breast cancer (all *P*<0.05), but other factors had no significant difference with the survival rate (Table 9).

**Table 9:** Multivariate survival analysis of OS using Cox’s regression model for breast cancer patients

Genotypes	B	S.E.M.	OR	95% CI	*P*
*EZH2* rs12670401	1.004	0.277	2.729	1.586–4.695	<0.001
*EZH2* rs6464926	0.928	0.275	2.529	1.474–4.339	0.001
*SMYD3*VNTR	0.358	0.184	1.431	0.998–2.051	0.052
*EZH2* mRNA	0.463	0.219	1.589	1.034–2.440	0.035
*SMYD3*mRNA	0.614	0.198	1.848	1.253–2.726	0.002
EZH2 protein	0.574	0.198	1.776	1.205–2.617	0.004
SMYD3 protein	0.495	0.191	1.640	1.128–2.385	0.010
Age	−0.172	0.528	0.842	0.299–2.372	0.745
Age of menarche	0.158	0.205	1.171	0.783–1.750	0.443
Menopausal status	0.594	0.471	1.810	0.720–4.553	0.207
Tumor size	0.012	0.142	1.012	0.767–1.336	0.931
ER (+)	−0.247	0.766	0.781	0.174–3.504	0.747
PR (+)	0.319	0.832	1.376	0.269–7.034	0.701
Histology	−0.049	0.151	0.952	0.708–1.281	0.746
Clinical staging	1.193	0.237	3.298	2.072–5.249	<0.001
Lymph node metastasis	0.742	0.223	2.101	1.358–3.251	0.001
HER2 status	1.204	0.255	3.335	2.024–5.494	<0.001
FDRs with breast cancer	−0.125	0.190	0.883	0.608–1.282	0.513
Subtypes of breast cancer	−0.088	0.287	0.915	0.522–1.606	0.758
Metastasis situation	0.668	0.189	1.950	1.345–2.826	<0.001

Abbreviation: B, regression coefficient.

## Discussion

The present study aims to explore the correlations of *EZH2* and *SMYD3* gene polymorphisms with breast cancer susceptibility and prognosis. Our findings suggest that *EZH2* rs12670401 and *EZH2* rs6464926 polymorphisms may be significantly correlated with breast cancer susceptibility and prognosis.

Our study found that C allele of *EZH2* rs12670401, T allele of *EZH2* rs6464926, and 3 allele of *SMYD3* VNTR could increase the susceptibility to breast cancer. *EZH2* gene, a histone methyltransferase gene and an important member of polycomb group (PcG), has been reported to be closely related to the formation and development of a variety of primary tumors (breast cancer included) and can play a role in the regulation of PcG through construction of polycomb-repressive complex 2 (*PRC2*) and in the modification of epigenetic genes [11,19]. *EZH2* rs12670401 polymorphism is located in the intron region of EID combined with EED, and *EZH2* rs6464926 is located in intron region of D2 combined with *SUZ12* (PcG protein). *PRC2* was formed by the combination of *EZH2* with *EED* and *SUZ12* that could activate histone methyltransferase enzyme [20–23]. Therefore, the polymorphism of the above loci may indirectly affect the combination of *EZH2* with *EED* and *SUZ12*, which may influence the histone methyltransferase function of *EZH2* so as to affect the function of histone in chromosomal methylation of *EZH2* [24–26]. *EZH2* as the subunit of the *PRC2* complex catalyzes trimethylation of histone H3 lysine 27 (H3K27) [27]. It is reported that the methylation level of H3K27 is closely related to the occurrence and prognosis of tumors [28]. Therefore, we concluded that the polymorphisms of *EZH2* may influence H3K27 methylation so as to function in the occurrence and prognosis of breast cancer. Besides, our results revealed that EZH2 and SMYD3 were in high expression in breast cancer tissues and cells. EZH2, an inhibitor of gene transcription, was related to biological malignancy in various cancers [29]. A previous study also found that high expression of EZH2 was associated with poor outcome in breast cancer [30]. SMYD3, a histone methyltransferase, played an important role in transcriptional regulation in human carcinogenesis [31]. High SMYD3 expression was critical for the development of breast cancer cells [32].

Unfortunately, we failed to reveal any correlations between *SMYD3* VNTR polymorphism and breast cancer susceptibility and prognosis. *SMYD3*, a protein with histone methylation function that could accelerate the methylation of chromosomes histone, is associated with the transcriptional cell regulation and presents high expression in a variety of tumors (breast cancer included) [32]. It has been reported that there exist differences in polymorphism of VNTR in the 5′ regulatory region of *SMYD3*, which might be closely related to the individual differences in the occurrence of tumorigenesis [16,17]. The 5′ regulatory region of *SMYD3* is a binding site of transcription factor *E2F-1* and plays an important role in cell cycle regulation and the occurrence of tumors*.* The increase in *E2F* in copy number of the region could enhance the affinity of *SMYD3* and E2F-1 and increase the possibility in occurrence of breast cancer and poor prognosis [16]. Thus, we hypothesized that *SMYD3* gene polymorphisms may be involved in the development and progression of breast cancer through combining with other genes and environmental factors.

Our study also found that the combined genotype of *EZH2* rs12670401 (TC + CC) and *EZH2* rs6464926 (CT + TT) could result in an obvious increase in the susceptibility to breast cancer, indicating that there may be interactions between the two loci. *EZH2* rs12670401 and *EZH2* rs6464926 polymorphisms have been proved to be in significant correlation with breast cancer susceptibility and prognosis [33]. The interaction mechanism of *EZH2* rs12670401 and *EZH2* rs6464926 polymorphisms in the development and progression of breast cancer is needed to be further studied in the future. Besides, we found that the age of menarche and menopause status had a certain relationship with breast cancer susceptibility and prognosis as well, which was consistent with a former study [34].

Another significant finding was that OS of patients with TT genotype was higher than those with TC + CC genotype of *EZH2* rs12670401 and OS of patients with CC genotype was higher than those with CT + TT genotype of *EZH2* rs6464926. The OS of patients with EZH2 high expression and SMYD3 high expression was lower than those with EZH2 low expression and SMYD3 low expression. Result of Cox multivariate analysis showed that *EZH2* rs12670401, *EZH2* rs6464926 and clinical staging, mRNA and protein expression of EZH2 and SMYD3, lymph node metastasis, HER2 status, and metastasis situation were independent prognostic factors for survival rate of breast cancer patients. EZH2 plays varied roles in cancer development relying on the type of cancers [35]. EZH2 expression is related to the OS of cancer patients and high EZH2 expression as a prognostic factor shows shorter OS for patients with breast cancer [36]. The T allele of *EZH2* 148505302 gene is associated with a longer OS in cholangiocarcinoma patients [37].

To summarize, our study provided strong evidence that *EZH2* rs12670401 and rs6464926 polymorphisms may be correlated with breast cancer susceptibility and prognosis. However, *SMYD3* VNTR polymorphism exhibited no association with breast cancer susceptibility and prognosis. Our findings provide a theoretical basis for breast cancer susceptibility assessment, breast cancer therapy, and clinical reference for personalized therapy of breast cancer. But the mechanism in the susceptibility to and survival of breast cancer remained unclear and therefore constant follow-up research is required.

## Abbreviations

95% CI: 95% confidence interval
CRC: colorectal cancer
DMEM: Dulbecco’s modified Eagle’s medium
ER^+^: estrogen receptor
*EZH2*: enhancer of Zeste homolog 2
FDRs: first-degree relatives
GAPDH: glyceraldehyde phosphate dehydrogenase
H3K27: histone H3 lysine 27
HER2: human epidermal growth factor receptor-2
IDC: invasive ductal carcinoma
ILC: invasive lobular carcinoma
OD: optical density
OR: odds ratio
OS: overall survival
PcG: polycomb group
PR^+^: progesterone receptor
*PRC2*: polycomb-repressive complex 2
RT-qPCR: reverse-transcription quantitative polymerase chain reaction
*SMYD3*: SET and MYND domain containing 3
